# RECRUITMENT STRATEGIES IN LONG-TERM CARE: NEW AND NOVEL APPROACHES

**DOI:** 10.1093/geroni/igae098.3000

**Published:** 2024-12-31

**Authors:** Cathy Maxwell, Judith Tate, Lisa Juckett, Kelley Colopietro, Abbey Kilpatrick, Lorraine Mion, Nilanjan Sarkar

**Affiliations:** University of Utah College of Nursing, Salt Lake City, Utah, United States; The Ohio State University, Columbus, Ohio, United States; The Ohio State University Medical Center, Columbus, Ohio, United States; Vanderbilt University, Nashville, Tennessee, United States; The Ohio State University, Columbus, Ohio, United States; The Ohio State University, Columbus, Ohio, United States; Vanderbilt University, Nashville, Tennessee, United States

## Abstract

Older adults residing in long term care (LTC) can benefit from social and behavioral research to improve health outcomes and promote evidence-based practice; however, recruitment is often difficult and time/cost-intensive. Systematic reviews of literature report recruitment barriers, as well as specific recruitment strategies. This presentation extends this work by reporting on new outreach endeavors within two state-based regions with over 300 LTCs. Our work is based on three longitudinal studies in LTC facilities to promote engineering and technology-based interventions that address apathy and loneliness in LTC residents. Relationship-building is the core of successful recruitment with establishment of trust and altruistic outreach. Our strategies include: 1) utilization of personnel with longstanding presence in the community and associated organizations (e.g., Alzheimer’s Association), 2) outreach opportunities to regional, state-based health care professional organizations, 3) repeated phone contacts to gauge interest, 4) provision of health screenings for residents (e.g., blood pressure, fall risk assessment), and 5) educational and enjoyable activities for residents (i.e., games, health-related topics [medications, nutrition, mind/body health, safety]). Research opportunities include studies using socially-assistive robots to address apathy among older adults with mild to moderate cognitive impairment, and augmented reality/avatar-based interventions to address loneliness among LTC residents. Our strategies established trust and promoted relationship-building between local and regional LTC facilities and the specific universities, resulting in successful recruitment of 17 (45%) out of 37 LTCs who responded to initial contact.

**Conclusion:**

Successful recruitment in LTC requires multiple strategies. Trust and relationship-building promote reciprocity that can benefit both LTC facilities and universities.

